# Investigation of the current situation regarding diagnosis and treatment of Alport syndrome in Asian countries: results of survey of the Asian Paediatric Nephrology association (AsPNA) tubular and inherited working group

**DOI:** 10.1007/s10157-023-02358-6

**Published:** 2023-06-08

**Authors:** Kandai Nozu, Lourdes Paula Real Resontoc, Nakysa Hooman, Anil Vasudevan, Jie Ding, Hee Gyung Kang

**Affiliations:** 1 Department of Pediatrics, Kobe University Graduate School of Medicine, 7-5-1 Kusunoki-Cho, Chuo, Kobe, Hyogo 650-0017 Japan; 2 Division of Pediatric Nephrology, College of Medicine, University of the Philippines-Philippine General Hospital, Manila, Philippines; 3 Ali Asghar Clinical Research Development Center, Department of Pediatrics, School of Medicine, Iran University of Medical Sciences, Tehran, Iran; 4 Department of Pediatric Nephrology, St John’s Medical College Hospital, Bengaluru, India; 5 Department of Pediatrics, Peking University First Hospital, Beijing, China; 6 Department of Pediatrics, Seoul National University Children’s Hospital, Seoul, South Korea

**Keywords:** Asia, Child, Nephritis, Hereditary, Alport syndrome, RAS inhibitors

## Abstract

**Background:**

Alport syndrome is one of the most common inherited kidney diseases worldwide. A genetic test or kidney biopsy is necessary for a definite diagnosis of this disease, and an accurate diagnosis system for this disease is highly desired in each country. However, the current situation in Asian countries is not clear. Therefore, the tubular and inherited disease working group of the Asian Pediatric Nephrology Association (AsPNA) aimed to assess the current situation of diagnosis and treatment for Alport syndrome in Asia.

**Methods:**

The group conducted an online survey among the members of AsPNA in 2021–2022. Collected data included the number of patients for each inheritance mode, availability of gene tests or kidney biopsy, and treatment strategies for Alport syndrome.

**Results:**

A total of 165 pediatric nephrologists from 22 countries in Asia participated. Gene test was available in 129 institutes (78%), but the cost was still expensive in most countries. Kidney biopsy was available in 87 institutes (53%); however, only 70 can access electron microscopy, and 42 can conduct type IV collagen α5 chain staining. Regarding treatment, 140 centers use renin-angiotensin system (RAS) inhibitors (85%) for Alport syndrome patients.

**Conclusions:**

This study result might suggest that the system is underdeveloped enough to diagnose all Alport syndrome patients in most Asian countries. However, once diagnosed with Alport syndrome, most of them were treated with RAS inhibitors. These survey results can be used to address knowledge, diagnostic system, and treatment strategy gaps and improve the Alport patients’ outcomes in Asian countries.

**Supplementary Information:**

The online version contains supplementary material available at 10.1007/s10157-023-02358-6.

## Introduction

Recently, the natural history of Alport syndrome has been reported, including registry-based and cross-sectional studies [1-5]. Most of them are from European countries, America, and part of Asian countries, including Japan, China, and Korea. In those countries, a comprehensive gene testing system for this disease has already been established, so accurate diagnosis of this disease is relatively easy. For the diagnosis of Alport syndrome, either a gene test or kidney biopsy is necessary [6]. In addition, for the pathological diagnosis, we need to find either abnormal glomerular basement membrane (GBM) changes, such as diffuse basket-weave changes by electron microscopy or abnormal expression of type IV collagen α5 chain (α5(IV)) by immunohistochemistry.

Recently, it has been clarified from both prospective and retrospective studies that renin-angiotensin system (RAS) inhibitors treatment can reduce the urinary protein level and delay the development of end-stage kidney disease (ESKD) remarkably [5, 7-9]. Therefore, an accurate and early diagnosis system for this disease is highly desired in each country to improve the prognosis of patients with Alport syndrome. However, the current situation in Asian countries is not clear. Therefore, the tubular and inherited disease working group of the Asian Pediatric Nephrology Association (AsPNA) aimed to assess the current status of diagnosis and treatment for Alport syndrome in Asian countries.

We have previously conducted a survey of the current status of pediatric tubular and inherited disorders in Asia as a first step [10]. The results highlight the diversity of disease prevalence, diagnostic practices, capability, and access to genetic tests across Asia. This time, we conducted the survey only for Alport syndrome to reveal the availability of a genetic test and kidney biopsy, including access to electron microscopy and α5(IV) staining. In addition, we asked the doctors about the treatment strategies. The data gathered from this survey can be used to address knowledge gaps and improve management and outcomes in Asian countries.

## Methods

### Survey development and conduct

We developed a web-based survey that included the following information (Supplementary data 1): (1) total patient numbers of Alport syndrome in each inheritance mode following at each institute. (2) Accessibility of gene test. If yes, we also asked about the cost of the gene test. (3) Availability of kidney biopsy. If yes, we also asked about the accessibility to electron microscopy and α5(IV) staining availability. (4) Treatment strategies, especially RAS inhibitor treatment. Using the Google platform or its equivalent for China, the final survey was administered by sending the link using the valid e-mail addresses of members of the AsPNA from October 2021 to April 2022. In centers with more than one nephrologist, we asked them to reply to only one representative doctor. Completion of the survey implied consent to participation. The survey closed on the last day of April 2022. Respondents did not receive remuneration for participating in this survey.

### Ethics

This study did not require human subjects or sensitive data covered by the data privacy act; we sought for waiver of consent.

## Results and discussion

As for the first survey covering many kinds of inherited kidney diseases, the results highlight the diversity of disease prevalence, diagnostic practices, capability, and access to genetic tests across Asia [10]. This second survey which focused on Alport syndrome again revealed the diversity of diagnostic strategies for this disease.

We received responses from 165 pediatric nephrologists and 165 institutes (academic; 110, public; 31, and private; 24) from 22 Asian countries (Table 1). Japan (*n* = 55), China (*n* = 40), and India (*n* = 14) have the most significant number of survey participants. In total, 1240 Alport syndrome patients were followed up in these institutes; 532 were male X-linked Alport syndrome (XLAS), 322 were female XLAS, 105 were autosomal recessive Alport syndrome (ARAS), and 84 were autosomal dominant Alport syndrome (ADAS).

**Table 1:** Data on the number of responding facilities and Alport syndrome in Asian countries

Country	Institute number answered the survey	Total number	XL (Male)	XL (Female)	AR	AD	Unknown	Gene test						Kidney biopsy			Treatment			
								Availability (%)	In-house test	Different center	Foreign countries	Genetic first approach	Average cost (USD)	Kidney biopsy availability (%)	Type 4 collagen staining	Electron microscopy	RAS treatment (%)	Other medicine	Medicine	RAS for Suspected cases (%)
Bangladesh	3	19	4	0	0	0	15	3 (100)	0	0	3	1	600	3 (100)	1	0	3 (100)	1	Cyclosporine(1)	2
China	40	501	225	140	25	16	14	34 (85)	12	22	0	31	700	14 (35)	10	13	34 (85)	8	Traditional chinese medicine(4), Cyclosporine(2), SGLT2(2), Spironolacton(1)	7
Hong Kong, China	1	10	7	0	1	2	0	1 (100)	N/A	N/A	N/A	1	N/A	1 (100)	1	1	1 (100)	0		0
India	14	41	35	2	5	5	7	5 (36)	0	5	0	4	215	12 (86)	4	11	14 (100)	0		3
Indonesia	1	0	0	0	0	0	0	0 (0)	0	0	0	0	-	1 (100)	0	0	0 (0)	0		1
Iran	4	84	24	9	8	12	9	2 (50)	1	1	1	0	N/A	3 (33)	1	3	3 (75)	1	Cyclosporine(1)	3
Japan	55	314	126	124	14	27	24	50 (91)	1	50	0	22	N/A	23 (42)	18	22	49 (89)	1	SGLT2(1)	2
Jordan	2	10	4	1	4	1	0	1 (50)	0	0	1	1	Free or 300	2 (100)	0	2	2 (100)	0		2
Korea	13	155	74	30	33	3	15	13 (100)	4	9	0	7	776	4 (31)	3	4	12 (92)	0		2
Laos	1	0	0	0	0	0	0	1 (100)	0	0	1	0	1300	0 (0)	0	0	1 (100)	0		1
Lebanon	2	2	0	0	0	0	2	1 (50)	0	0	1	1	650	1 (50)	1	1	1 (50)	0		1
Malaysia	4	10	4	2	2	3	3	4 (100)	0	0	4	4	438	3 (75)	0	2	4 (100)	0		2
Mongolia	2	48	11	7	7	11	13	2 (100)	0	0	2	1	100	2 (50)	1	2	2 (100)	1	Dipyridamole, Ascorutin, Vit A,E	2
Myanmar	2	2	0	0	0	0	0	1 (50)	0	0	1	0	2000	2 (100)	0	0	1 (50)	0		0
Nepal	3	6	1	0	0	0	5	2 (67)	0	0	2	0	350	3 (100)	0	0	2 (67)	0		2
Pakistan	3	9	4	1	1	3	4	1 (33)	0	0	1	0	N/A	3 (100)	0	1	2 (67)	0		2
Philippines	8	6	1	2	1	0	4	3 (38)	0	0	3	0	free or 400	6 (75)	1	6	3 (38)	0		6
Sri Lanka	2	3	0	2	0	0	1	0 (0)	0	0	0	0	–	2 (100)	0	0	1 (50)	0		2
Thailand	1	0	0	0	0	0	0	1 (100)	0	1	0	0	N/A	1 (100)	0	0	1 (100)	0		0
UAE	3	10	4	1	4	1	1	3 (100)	0	0	3	2	1017	3 (100)	2	3	3 (100)	0		1
Vietnam	1	10	8	1	0	0	1	1 (100)	0	0	1	0	N/A	0 (0)	0	0	1 (100)	0		0
Total	165	1240	532	322	105	84	118	129	18	88	24	75		89	43	71	137 (83)	12		41 (25)

Although most of the institutes (78%) had access to gene tests in Asian countries, most of them sent genomic DNA samples to foreign countries except for China, India, Iran, Japan, Korea, and Thailand. In that case, gene tests are costly (100–2000 USD). The ‘genetic first approach’ was defined as a diagnostic approach of directly conducting gene tests before kidney biopsy when physicians see patients with hematuria and a family history of chronic kidney diseases (CKD). This is a common approach among responders in Malaysia, China and Japan.

As for kidney biopsy, it is available in about half of the institutes (54%) in Asian countries; however, most of the institutes, except for some countries, do not have access to type IV collagen α5 staining (26%) or electron microscopy (43%). These two items are indispensable for the pathological diagnosis of Alport syndrome without which a definite pathological diagnosis of this disease is impossible [6].

Regarding treatment, almost all pediatric nephrologists use renin-angiotensin system (RAS) inhibitors. Some of them still use cyclosporine, which is currently considered more harmful than beneficial in this disease [11, 12]. Doctors in Asian countries tend to hesitate to start RAS inhibitor treatment for suspected but not definitely diagnosed Alport syndrome patients. To the consensus of our working group, patients with hematuria and proteinuria who are not yet diagnosed should be started RAS inhibitor treatment because it benefits the patients more than harms them.

Of course, this study has many limitations that must be addressed. 1. The survey only included members of the AsPNA, and thus it may have yet to capture the actual burden of the diseases in Asia. However, it was very difficult to obtain detailed data such as what percentage of pediatric nephrologists in each country have answered the survey. 2. We asked separately for the patient number and availabilities for gene tests and kidney biopsy. So we still need to get the information on how many patients were diagnosed by these tools. It means the proportion of the inheritance mode needs to be more reliable. 3. Although most of the institutes have access to gene tests, it is expensive, and only some suspected patients can have gene tests, but we have yet to ask what proportion of the patients have conducted gene tests. Therefore, in the future survey, we should have revealed how many patients are correctly diagnosed based on genetic tests. 4. We have not yet investigated the precise method of RAS inhibitor treatment for Alport syndrome patients. In a further study, we need to know the age of start taking RAS inhibitors and their efficacy.

Clinical practice guidelines published in Western countries are not always applicable to Asians. The most significant difference with Western countries is the complete lack of diagnostic systems in most Asian countries. Fortunately, RAS inhibitors are not expensive medicines, and may be recommended to start these drugs even for suspected Alport syndrome patients but not a definitive diagnosis because the benefits of treatment with these agents outweigh the drawbacks to these patients. Therefore, Asia-specific clinical practice guidelines should be developed for rapid, correct diagnosis and treatment to improve disease outcomes suitable to this particular group. The aim of this study was to solve the knowledge gap and improve the management and outcomes of Alport syndrome in Asia. To bridge the gaps in the knowledge of the disease, diagnostic practices, and access to genetic tests, a network for broader collaborations is needed in undertaking prospective research studies. Free webinars for these countries should be planned by AsPNA.

In conclusion, the results highlight that the availability of gene tests or kidney biopsies for diagnosing Alport syndrome is needed in Asian countries. This result might suggest that the system is underdeveloped enough to save all Alport syndrome patients. However, once diagnosed with Alport syndrome, most of them were treated with RAS inhibitors. These survey results can be used to address knowledge, diagnosis system, and treatment strategy gaps and improve the Alport patients’ outcomes in Asian countries.

## Supplementary Information

Below is the link to the electronic supplementary material.

Supplementary file1 (XLSX 36 KB)

Supplementary file2 (PDF 486 KB)
