# Supplementary material for: Investigation of the current situation regarding diagnosis and treatment of Alport syndrome in Asian countries: results of survey of the Asian Paediatric Nephrology association (AsPNA) tubular and inherited working group
Source: Clin Exp Nephrol. 2023 Jun 8;27(9):776–80. doi: 10.1007/s10157-023-02358-6 (PMC10432360; doi:10.1007/s10157-023-02358-6)
Supplement: Supplementary file 2 — Supplementary file2 (PDF 486 KB) [file 10157_2023_2358_MOESM2_ESM.pdf]

Asian Pediatric Nephrology Association (AsPNA) Tubular and Inherited Disorders Working Group

The purpose of this study is to clarify how patients with Alport syndrome are diagnosed and treated in Asian countries. In recent years, it has become clear that ACE inhibitors (ACEI) and Angiotensin receptor blockers (ARB) are very effective for this disease and can delay the development of End-stage kidney disease (ESKD). We will analyze the data with the aim of reducing the disparities between countries regarding the diagnosis and treatment of patients with this disease and so that they can receive better treatment. Only one pediatric nephrologist from each facility should aggregate the data for that facility and answer this survey. You do not need to include patients' data from adult section or already transferred to adult section.
Thank you for your cooperation.

* Required

1. Email *

____

2. 1. Name

____

3. 2. Country *

____

4. 3. Institution (Check all that apply) *

Check all that apply.

- [ ] Academic
- [ ] Research
- [ ] Government-funded
- [ ] Private
- [ ] Non-academic
- [ ] Non-research

5. 4. Name of Hospital *

6. 5. Your age category? *

Mark only one oval.

- 18-24
- 25-34
- 35-44
- 45-54
- 55-64
- 65+
- Other: ____________

7. 6. How many Alport syndrome PATIENTS and FAMILIES are you following in your department?

8. 7. Please specify the number of x-linked MALE Alport syndrome patients in your care

9. 8. Please specify the number of x-linked FEMALE Alport syndrome patients in your care

10. 9. Please specify the number of autosomal dominant Alport syndrome patients in your care

11. 10. Please specify the number of autosomal recessive Alport syndrome patients in your care

____

12. 11. Please specify the number of Alport syndrome patients with UNKNOWN inheritance pattern in your care.

____

13. 12. What was the diagnostic criteria for these patients/families?(Can have multiple answers)

Check all that apply.

- [ ] Gene test
- [ ] Pathology (Kidney biopsy)
- [ ] Pathology (Skin biopsy)
- [ ] Clinical features and family history

14. 13. For diagnosis using pathology, indicate specific tests done (Check all that apply)

Check all that apply.

	Light microscopy	Electron microscopy	type 4 collagen α5 chain staining
**Kidney biopsy**	☐	☐	☐
**Skin biopsy**	☐	☐	☐

15. 14. Does your institute have access to gene test? If NO, proceed to Section 2.

Mark only one oval.

- ◯ Yes
- ◯ No

16. 15. Is it an in-house gene test system?

Mark only one oval.

- ( ) Yes
- ( ) No

17. 16. If it's not an in-house gene testing, where do you send your specimen?

Mark only one oval.

- ( ) Different center but in your own country
- ( ) Foreign country

18. 17. How many percent of your patients are diagnosed using gene test?

____

19. 18. Do you conduct gene test prior to kidney biopsy when patients are having hematuria and proteinuria, and familial history of CKD?

Mark only one oval.

- ( ) Yes
- ( ) No

20. 19. Billing or payment for gene test

Check all that apply.

	Yes	No
**Free of charge**	☐	☐
**Health Insurance**	☐	☐
**Charged to patients (out-of-pocket)**	☐	☐

21. 20. If charged to patients? How much does it cost? (in USD)

22. 21. When Alport syndrome patients show both proteinuria and hematuria, do you routinely start the treatment by ACE inhibitors (ACEI) or angiotensin receptor blockers (ARB)?

Mark only one oval.

- Yes. To almost all cases.
- Yes, but only for male cases with X-linked Alport syndrome.
- No
- Other:

23. 22. Do you routinely treat Alport syndrome patients with medicines other than ACEI/ARB?

Mark only one oval.

- Yes
- No

24. 23. If “Yes”, please describe what kind of medicines do you use.:

For institutions with no access to gene test and/or kidney biopsy

25. 1. Do you have familial cases with CKD accompanied by hematuria and suspected with Alport syndrome, but because you do not have an access to gene test, you can't make the definitive diagnosis of Alport syndrome

Mark only one oval.

- ⬭ Yes
- ⬭ No

26. 2. How many patients/families do you have?

____

27. 3. Do you have an access to kidney biopsy (including center or core hospitals you can refer your patients to)?

Mark only one oval.

- ⬭ Yes
- ⬭ No

28. 4. For kidney biopsy

Check all that apply.

	Yes	No
**Type 4 collagen α5 chain staining**	☐	☐
**Electron microscopy**	☐	☐
**Free of charge**	☐	☐
**Full coverage by health insurance**	☐	☐
**Partial coverage by health insurance**	☐	☐
**Out-of-pocket (Patients pay for the test)**	☐	☐

29. 5. If out-of-pocket, how much does the kidney biopsy cost? (in USD)

____

30. 6. If partial coverage by health insurance, how much does the patient have to pay? (in USD)

____

31. 7. When Alport syndrome patients show both proteinuria and hematuria, do you routinely start the treatment by ACE inhibitors (ACEI) or angiotensin receptor blockers (ARB)?

Mark only one oval.

- Yes. To almost all cases.
- Yes, but only for male cases with X-linked Alport syndrome.
- No
- Other: _______________________________

32. 8. Do you start treatment by ACEI/ARB when you see cases with hematuria and proteinuria, and familial history of ESKD, but you do not have an access to gene test and/or kidney biopsy.

Mark only one oval.

- Yes
- No

33. 9. Do you routinely treat Alport syndrome patients with medicines other than ACEI/ARB?

Mark only one oval.

- Yes
- No

34. 10. If “Yes”, please describe what kind of medicines do you use.

This content is neither created nor endorsed by Google.

Google Forms
